# Testing Usability and Feasibility of a Mobile Educator Tool for Pediatric Diabetes Self-Management: Mixed Methods Pilot Study

**DOI:** 10.2196/16262

**Published:** 2020-05-01

**Authors:** Marisa Otis, Jack Zhu, Suleiman N Mustafa-Kutana, Angelina V Bernier, Julio Ma Shum, Arlette A Soros Dupre, Monica L Wang

**Affiliations:** 1 Department of Community Health Sciences Boston University School of Public Health Boston, MA United States; 2 Division of Pediatric Endocrinology and Metabolism Boston Medical Center Boston, MA United States; 3 Division of Pediatric Endocrinology University of Florida Gainesville, FL United States

**Keywords:** diabetes mellitus, self-management, health education, mHealth, mobile health, child health

## Abstract

**Background:**

Mobile interventions hold promise as an intervention modality to engage children in improving diabetes self-management education, attitudes, and behaviors.

**Objective:**

This pilot study aimed to explore the usability, acceptability, and feasibility of delivering a mobile diabetes educational tool to parent-child pairs in a clinical setting.

**Methods:**

This mixed methods pilot study comprised two concurrent phases with differing study participants. Phase 1 used user testing interviews to collect qualitative data on the usability and acceptability of the tool. Phase 2 used a single-arm pre- and poststudy design to quantitatively evaluate the feasibility and preliminary efficacy of the intervention. Study participants (English-speaking families with youth aged 5-14 years with insulin-dependent diabetes) were recruited from an urban hospital in Massachusetts, United States. In phase 1, parent-child pairs were invited to complete the intervention together and participate in 90-min user testing interviews assessing the tool’s usability and acceptability. Interview transcripts were analyzed using a directed content analysis approach. In phase 2, parent-child pairs were invited to complete the intervention together in the clinical setting. Measures included parental and child knowledge, attitudes, and behaviors related to diabetes management (self-report surveys) and child hemoglobin A1c levels (medical record extractions); data were collected at baseline and 1-month follow-up. Pre- and postoutcomes were compared using paired t tests and the Fisher exact test.

**Results:**

A total of 11 parent-child pairs (N=22) participated in phase 1 of the study, and 10 parent-child pairs (N=20) participated in phase 2 of the study. Participants viewed the mobile educational tool as acceptable (high engagement and satisfaction with the layout, activities, and videos) and identified the areas of improvement for tool usability (duration, directions, and animation).

**Conclusions:**

The findings from this pilot study suggest that the mobile educational tool is an informative, engaging, and feasible way to deliver diabetes self-management education to parents and children in an urban hospital setting. Data will inform future iterations of this mobile diabetes educational intervention to improve usability and test intervention efficacy.

## Introduction

### Background

Type 1 diabetes (T1D) and type 2 diabetes (T2D) are among the most common chronic illnesses in children. Rates of T2D are disproportionately higher among American Indian, Hispanic, and black youth compared with white youth in the United States [1]. Pediatric diabetes adversely affects quality of life, productivity, and life expectancy and contributes to enormous health care costs, particularly given its increasing prevalence [2-5]. Earlier onset of diabetes also increases the risk and severity of diabetes-related complications [6,7]. Developing strategies to help youth and parents succeed in diabetes self-management is needed to promote positive health outcomes.

Extensive education is required to equip youth and families with the knowledge and skills needed to manage diabetes. Current diabetes management educational tools provided to families in the clinical setting are inadequate and text heavy, with parents often reporting that the educational process is overwhelming and lacks a patient-centered approach [8-10]. Furthermore, current diabetes self-management educational materials are not child centered (developed based on the needs and interests of children). As children transition into adolescence, the responsibility for diabetes management shifts from parents to the children [11]. Educational materials designed specifically for children, particularly those in preadolescence and early adolescence, are needed to facilitate the transition from parent-initiated diabetes management to self-management. To ease this transition, educational materials need to (1) engage youth and (2) facilitate positive, productive communication between parents and youth [12].

A growing number of studies have used mobile health to target diabetes self-management among adolescents [10,13-17]. The findings from such studies indicate the potential for mobile health interventions to enhance diabetes education, motivate behavior change, and have widespread dissemination. However, prior studies on mobile diabetes self-management interventions have focused primarily on adolescents (aged 12 to 19 years) [15-17]. The extent to which mobile diabetes self-management tools may be applicable for younger children and facilitate productive parent-child communication has not been extensively explored. In-depth research with parents’ and children’s perceptions and experiences with a child-centered, mobile diabetes educational intervention can inform the development and improvement of such tools.

### Objectives

This pilot study aimed to (1) gather qualitative data on the usability and acceptability of the Mobile Diabetes Educator (MDE) prototype through user testing interviews with parent-child pairs (phase 1) and (2) assess the feasibility and preliminary efficacy of delivering the MDE in a clinical setting among parent-child pairs through a single-arm pre- and posttrial (phase 2). Phases were run concurrently with differing study participants using a mixed methods approach to achieve study aims. We hypothesized that trends in improvements in diabetes knowledge, attitudes, and self-management behaviors at 4- to 6-week follow-up would be observed.

## Methods

### Mobile Diabetes Educator Intervention Prototype

**Figure 1:**
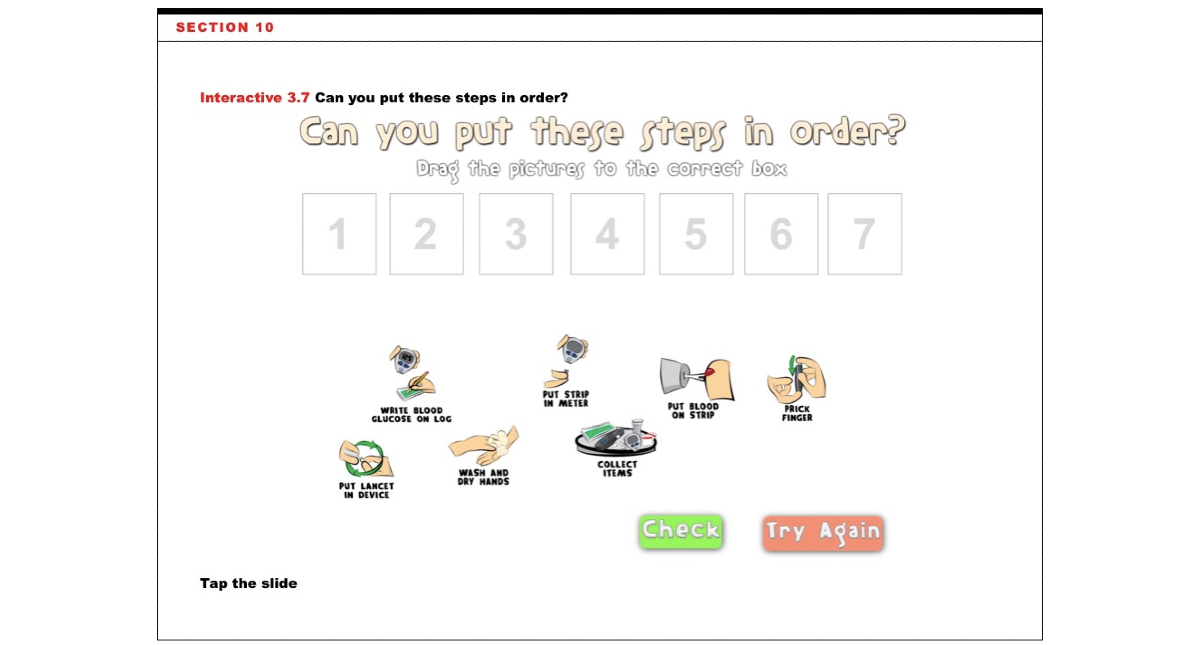
A page in the Mobile Diabetes Educator (MDE) that features interactive learning. This activity asks the patient to drag various steps needed for blood glucose monitoring into the correct order and check their results.

**Figure 2:**
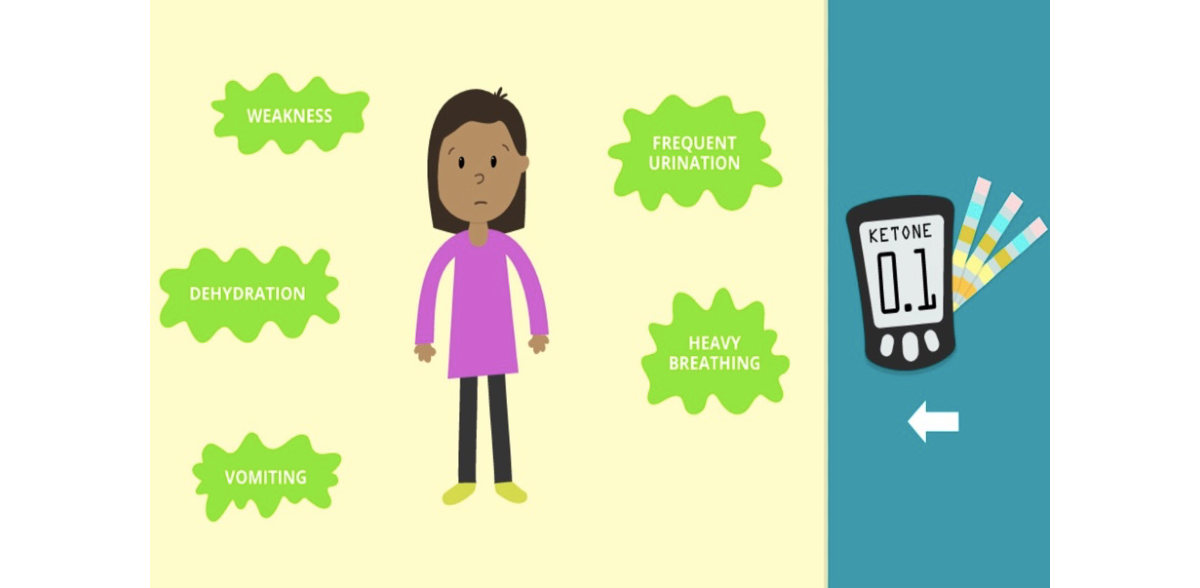
A page in the Mobile Diabetes Educator (MDE) that presents symptoms of hyperglycemia.

In collaboration with children’s educational media consultants, the study team developed a prototype of the MDE [18]. This mobile educational program (interactive electronic book) was designed for school-aged youth and intended to be navigated by parents and children together to facilitate communication. The MDE prototype consists of eight animated, interactive modules that feature an ethnically ambiguous preadolescent character named Kara who has T1D (Figures 1 and 2). Topics covered include diabetes etiology, managing glucose levels, and diet and exercise recommendations. Multimedia strategies (eg, images and videos) were used for information delivery as they play a crucial role in learning. Images serve to improve perception, understanding, and memory and to encourage engagement by the user [19,20]. Using visuals is also an important mode of risk communication because the visual cortex of the brain becomes activated during high-stress situations [21,22].

The tool can be accessed through any mobile or computer device with internet access, although it was specifically designed for touchscreen navigation on a tablet. The interactive modules were designed to be completed in segments, self-directed at the patient’s own pace, to replace text-based educational materials in the clinical setting after an initial diabetes diagnosis. For the purposes of this study, the modules were completed in one sitting (approximately 45-60 min).

### Study Participants and Setting

In both phases of the study, child participants and their parents or caregivers were recruited through the Pediatric Diabetes and Endocrinology Section at Boston Medical Center (BMC), a safety net urban hospital in Massachusetts, United States. A wide age range (elementary school–aged and middle school–aged youth) of child participants was used to examine the extent to which the tool was acceptable, engaging, and relevant across different age ranges. Eligibility criteria for child participants included (1) being aged 5 to 14 years, (2) being diagnosed by a clinician as insulin dependent (T1D or T2D), (3) currently receiving diabetes care at BMC, (4) parent consent to participate, and (5) being able to read and converse in English. Eligibility criteria for parent participants included (1) being aged 18 years or older and (2) being able to read and converse in English. All study procedures took place in the BMC clinical setting.

### Phase 1: User Testing Interviews and Measures

Phase 1 consisted of collecting qualitative data to assess usability and acceptability through user testing observations and interviews, an appropriate method given the low literacy level of our target population and the cognitive developmental stage of some of our child participants (eg, children aged 5-7 years may not understand how to answer standardized measures of usability, whereas study team observations and open-ended questions may generate more insights). User testing lasted approximately 90 min and consisted of an observational component and a semistructured interview. The study team provided participants with a tablet with the intervention preloaded and asked participants to use the tool and explain their thinking out loud as they navigated the tool. Study staff silently observed how participants navigated the tool and noted areas for improvement in *usability* (ease of navigation and problems encountered, time taken to complete the tool, and identification of the primary user [parent, child, or equal use between parents and children]) and *acceptability* (frequency, content, and tone of parent-child communication for pair users and level of participant engagement). Immediately after completing the tool, study staff conducted semistructured interviews and asked participants to evaluate the tool in terms of additional *acceptability* measures (clarity of content, acceptability of contexts [eg, characters and settings], perceived purpose of the tool, overall satisfaction, and areas for improvement; see Multimedia Appendix 1).

### Phase 1: Qualitative Analysis

Interviews were audio recorded, transcribed verbatim, and thematically analyzed by study staff. The analysis used a directed content analysis approach [23]. An initial codebook was developed based on the interview guide. Following transcript coding by study staff, codes were revised to incorporate additional themes as needed. Thus, both a priori and de novo themes were identified and given an operational definition. Coders also identified quotes that represented each theme.

### Phase 2: Feasibility Trial and Measures

Phase 2 consisted of collecting quantitative data to assess preliminary efficacy using self-reported surveys and medical record information. Parent and child participants completed a baseline assessment immediately before participating in a 1-hour intervention session and a postassessment 4 to 6 weeks later (all completed in the clinical setting). For the intervention session, parent-child pairs were provided with a tablet and asked to complete the intervention (self-administered) together. The following four measures on diabetes: knowledge, self-efficacy, self-management, and communication were each completed separately by both child and parent participants using self-report, self-administered surveys. *Diabetes knowledge* was assessed using 25 multiple-choice items from the Revised Brief Diabetes Knowledge Test [24], with the percentage of correctly answered items calculated. Sample diabetes knowledge topics covered included nutrition, glucose testing, glucose reactions, and insulin. Sample questions included (1) A low blood glucose reaction may be caused by too much insulin, too little insulin, too much food, or too little exercise and (2) Which of the following is highest in carbohydrates? Baked chicken, swiss cheese, baked potato, or peanut butter. *Diabetes management self-efficacy* was measured using 19 items from the Diabetes Self-Efficacy Scale [25], which asked respondents to rate their confidence in their ability to manage diabetes (eg, glucose self-monitoring, insulin injections, and meal planning) using a 5-point Likert scale, ranging from 1 (very sure I cannot) to 5 (very sure I can). *Perceived readiness for diabetes self-management* was measured using two items from the Readiness to Change the Balance of Responsibility Scale [26]. These items included “I feel ready to manage diabetes on my own” and “I feel ready to take on some, but not all, of diabetes management on my own,” which were assessed using a 5-point Likert scale, ranging from 1 (very sure I cannot) to 5 (very sure I can). The *frequency of parent-child diabetes management communication* was assessed using three items from the Self-Management of T1D in Adolescence subscale [27]. Participants were asked to report how often they talk to their parents about diabetes, when they have questions about diabetes, and when they have problems managing diabetes using a 4-point frequency scale (always, sometimes, occasionally, and never). Child sociodemographics included gender, age, race and ethnicity, and type of insulin-dependent diabetes diagnosis (T1D or T2D). Child hemoglobin A_1c_ (HbA_1c_) levels were extracted from child participants’ medical records by a trained patient navigator. Parental sociodemographics included gender, age, race and ethnicity, annual household income, highest level of education completed, and occupational status (employed full time, employed part time, or other).

### Phase 2: Statistical Analysis

Distributions, descriptive statistics, and missing values were examined for all measures. Changes in parent and child outcomes from pre- and postassessments were analyzed using paired *t* tests for continuous outcomes and the Fisher exact test for categorical outcomes. Statistical analyses were conducted using SAS version 9.4. The data were considered to be statistically significant at an alpha value of .05.

## Results

### Sociodemographics

The phase 1 study sample consisted of 11 parent-child pairs (N=22). Among child participants, the mean age was 10.3 (SD 2.2) years, and nearly two-thirds (7/11, 64%) of them were female. Almost all (10/11, 91%) participants had T1D. Among parents, the mean age was 39.5 (SD 11.0) years. The majority of parent participants were female (10/11, 91%), had less than a college degree (10/11, 91%), and had an annual household income less than US $50,000 (9/11, 82%). See Table 1 for additional sociodemographics on phase 1 participants.

**Table 1:** Baseline characteristics of the parent-child pairs participating in phase 1 and phase 2 of the Mobile Diabetes Educator pilot study (2018-2019).

Baseline characteristics				Phase 1 (N=11)		Phase 2 (N=10)
**Child**						
	**Gender, n (%)**					
		Female	7 (64)		5 (50)	
		Male	4 (36)		5 (50)	
	Age (years), mean (SD)		10.3 (2.2)		10.8 (2.9)	
	**Race, n (%)**					
		White	1 (9)		1 (10)	
		Black	6 (55)		5 (50)	
		Hispanic or Latino	1 (9)		1 (10)	
		Other	3 (27)		3 (30)	
	**Type of insulin-dependent diabetes, n (%)**					
		Type 1	10 (91)		9 (90)	
		Type 2	1 (9)		1 (10)	
**Parent**						
	**Gender, n (%)**					
		Female	10 (91)		8 (80)	
		Male	1 (9)		2 (20)	
	Age (years), mean (SD)		39.5 (11.0)		40.8 (11.2)	
	**Race, n (%)**					
		White	2 (18)		3 (30)	
		Black	8 (73)		6 (60)	
		Hispanic or Latino	1 (9)		1 (10)	
		Other	0 (0)		0 (0)	
	**Annual household income, n (%)**					
		Less than US $30,000	4 (36)		5 (50)	
		US $30,000-$49,999	5 (45)		3 (30)	
		Greater than or equal to US $50,000	2 (18)		2 (20)	
	**Education, n (%)**					
		Less than or equal to high school degree	6 (55)		6 (60)	
		Some college	4 (36)		3 (30)	
		Greater than or equal to college degree	1 (9)		1 (10)	
	**Occupation, n (%)**					
		Employed full time	6 (55)		4 (40)	
		Employed part time	2 (18)		2 (20)	
		Other (disabled, retired, unemployed, or homemaker)	3 (27)		4 (40)	

The phase 2 study sample consisted of 10 parent-child pairs (N=20). Among children, the mean age was 10.8 (SD 2.9) years, and half (5/10, 50%) of them were female. Almost all (9/10, 90%) participants had T1D. Among parents, the mean age was 40.8 (SD 11.2) years. The majority of parent participants were female (8/10, 80%), had less than a college degree (9/10, 90%), and had an annual household income less than US $50,000 (8/10, 80%). See Table 1 for additional sociodemographics on phase 2 participants.

### Phase 1: User Testing Observations

Study staff’s observations of parent-child interactions during user testing provided insight into the usability and acceptability of the MDE tool (Table 2). The majority (6/11, 55%) of parent-child pairs demonstrated shared use of the tool (ie, taking turns holding the tool, reading content, and completing activities), although in some (4/11, 36%) instances, the child was the primary user. Although most (9/11, 82%) users encountered problems using the tool, the overall navigation of the tool appeared either easy (5/11, 46%) or moderately easy (4/11, 36%) according to study staff’s observations. Parental and child engagement with the tool was distributed by engagement level. Observers noted that 55% (6/11) of parents and 36% (4/11) of children were highly engaged, whereas 27% (3/11) of parents and 36% (4/11) of children demonstrated a moderate level of engagement. Observers also noticed high (5/11, 46%) and moderate (3/11, 27%) parent-child communication during user testing.

**Table 2:** User testing observations of 11 parent-child pairs participating in phase 1 of the Mobile Diabetes Educator pilot study (2018-2019).

Staff-rated observations			Values, n (%)
**Ease of navigation**			
	Easy	5 (45)	
	Moderate	4 (36)	
	Difficult	2 (18)	
**User problems encountered**			
	Yes	9 (82)	
	No	2 (18)	
**Primary user**			
	Parent	1 (9)	
	Child	4 (36)	
	Equal use	6 (55)	
**Parent engagement**			
	High	6 (55)	
	Moderate	3 (27)	
	Low	2 (18)	
**Child engagement**			
	High	4 (36)	
	Moderate	4 (36)	
	Low	3 (27)	
**Parent-child communication**			
	High	5 (45)	
	Moderate	3 (27)	
	Low	3 (27)	

### Phase 1: User Testing Interviews

Thematic analysis of semistructured interviews with parent-child pairs further explored the usability and acceptability of the MDE tool. In total, seven themes were identified and coded based on the semistructured interview guide: usability, comprehension, high engagement, low engagement, purpose, satisfaction, and suggestions for improvement. These themes, alongside illustrative quotes, are summarized in Table 3 and discussed below.

**Table 3:** Illustrative quotes by theme from user testing interviews with 11 parent-child pairs participating in phase 1 of the Mobile Diabetes Educator pilot study (2018-2019).

Theme		Illustrative quotes
**Usability**		
	Somewhat user friendly	“I think certain areas were just a little confusing so you couldn’t really tell whether or not you’re supposed to tap on it or are you just supposed to go to the next screen.”
**Comprehension**		
	High comprehension	“It got to the point. It explained the situations and what to look for in the situations. It explained it a lot. A kid would understand it.”
**High engagement**		
	Layout	“I liked that it alternated with videos and then text.”
	Activities	“I think they are helpful in the sense that they kind of test your knowledge and help you to get a better understanding of it.”
	Chapters	“My favorite part was basically explaining why people have diabetes.”
	Videos	“I liked the animated videos. I think that keeps you going. Looking at the lady sitting there just talking [whereas] looking at the animation, they’re doing things so it makes you want to look at it more.”
**Low engagement**		
	Activities	“Some of them were confusing and some of them were boring. And then some of them were just ‘meh.’”
	Chapters	“Too many chapters.”
	Characters	“I just thought it was weird they had no arms and legs.”
**Purpose**		
	Health education	“To educate kids about diabetes in a fun way. To let them know it’s ok to have diabetes. This stuff happens in normal life, it happens in school. It tells them what to do also, but in a fun way.”
	Health management	“Learning about it and how to maintain it, and keep yourself healthy.”
	Motivation for diabetes self-management	“To tell us that it is not easy to take care of [diabetes] but you have to try your best and eat more healthy food so you won’t get sick.”
**Satisfaction**		
	Moderate satisfaction	“I say 7 [out of 10] because like I said from earlier, just those little kinks that need to be worked out. But outside of that, I think it’s a really good tool to educate and inform others about diabetes, especially for a child that is new to it and kind of clueless and going through it. So, it’s a good way to help them to understand it on their level.”
**Suggestions for improvement**		
	Usability	“A little more instruction at the top of the screen on what’s expected on that particular screen, that slide.”
	Comprehension	“I would only suggest doing the content a little bit more on the kids’ level–especially for younger kids–so they could really grasp the content just a little bit more. [For example], with the video with the nutrition, just doing all the way around on an animated level for kids if they’re going to be the ones engaging on the iPad. But if it’s more like older kids or adults, then yeah keep it the way it is.”
	Layout	“A little more videos and quizzes.”
	Chapters	“I think they should have added more about the carbs. They should have at least given more clarification on how you would...how much insulin and how many carbs...they should have put that together more.”
	Activities	“Drawing. Maybe have the kids draw their idea of the pancreas and all that stuff.”
	Characters	“I think she should sound more like a kid. I think all of them should.”
	Settings	“More settings would be nice. Especially when we’re taking trips out and then to know what you need or how to do things when you’re in a car. Stuff like that.”

#### Usability

Most participants described the tool as somewhat user friendly. Any difficulty using the tool was attributed to two main reasons: a lack of directions and malfunctioning activities. Given that there is a mix of text, videos, and activities throughout the tool, users felt unclear at times of what they were supposed to do on any given slide (eg, whether it was an interactive slide or not and what to press to engage in activity). Users also expressed frustration with some activities that did not work (eg, press button and nothing happens). However, in some cases, what appeared to be a malfunction was just difficulty navigating the activity because of a lack of directions (eg, unclear that the user must select icons sequentially for an activity to work).

#### Comprehension

Participants found the tool to be informative and thought the material was easy to understand, signifying an appropriate literacy level. Both parent and child users could demonstrate that they learned diabetes-related information after completing the tool. Notably, the visuals used throughout the tool (eg, pictorial representation of symptoms) helped increase comprehension of the information being conveyed.

#### High Engagement

As participants discussed parts of the tool they liked the most, several subthemes emerged, including layout, chapters, activities, and videos. The overall layout of the tool, which included a mix of text, videos, and activities, provided both passive and active learning opportunities for users, thereby increasing their engagement with the tool (eg, was able to hold children’s attention by giving them things to do). Participants largely felt that the chapters covered all the basic topics. The chapters on diabetes etiology and celebrities with diabetes were particularly engaging for children, whereas the chapters that demonstrated diabetes management and care (eg, insulin injection) were of interest to parents. The activities were often cited as the most engaging aspect of the tool and a positive way to reinforce the knowledge learned. Participants also enjoyed the videos, as they made the information easier to understand (eg, animated cells and organs were helpful in understanding diabetes etiology). Although participants liked the balance of animated and nonanimated videos, some cited the animations as slightly more engaging, especially for children.

#### Low Engagement

As participants discussed parts of the tool they found less interesting, the following subthemes emerged: duration, characters, and setting. The average time it took for users to complete the tool was 56 min (SD 15), which many felt was too long and contributed to a general feeling of boredom. Although participants typically liked the main character and her personality, they repeatedly expressed dissatisfaction with the robotic voice and appearance (eg, missing arms and legs) of the animated characters. Similarly, although the school-based settings of the animated videos felt true to life, participants would have liked to see more settings, particularly when children are on the go (eg, in the car) or without their parents (eg, birthday party and sleepover).

#### Purpose

Most participants thought the main purpose of the tool was to provide health education (eg, target knowledge). Participants described the target audience for this tool as newly diagnosed patients or people who do not already know about diabetes. Other main purpose subthemes that emerged included health management (eg, target behaviors) and motivation for diabetes self-management (eg, target attitudes).

#### Satisfaction

The majority of participants reported high satisfaction with the tool. On a scale of 1 to 10, with 10 being the best, participants’ average rating was 8.7 (SD 1.5). High satisfaction was often attributed to the fact that the tool was entertaining and provided a great deal of information. Factors that lowered users’ satisfaction scores included the long duration and, thus, boringness of the tool as well as the aforementioned usability issues with some activities.

#### Suggestions for Improvement

Participants suggested several ways to improve the tool in relation to the following subthemes: usability, comprehension, layout, activities, characters, duration, and dissemination. To improve the tool’s usability, clear directions should be included on each slide, and malfunctioning activities should be fixed. Comprehension could be enhanced by increasing the use of child-friendly explanations of information (eg, pictures to complement or replace words and more animation). Similarly, participants thought more engaging, creative features, such as videos and activities, should be added to enrich the overall layout of the tool. Suggestions for new activities included a word search, summary quiz, drawing, or having the user watch a video on how to perform a behavior and then practice doing so on a cartoon. According to participants, animated characters should have a less robotic voice and more realistic appearance. The use of superhero characters was also suggested. Finally, participants agreed that the duration of the tool should be shortened, yet they found it difficult to identify ways to do so (eg, information that could be cut). However, disseminating the MDE as an app was recommended to allow users to cover the material at their own pace at home.

### Phase 2: Outcomes

Participants spent an average of 59.2 (SD 5.4) min to complete the intervention in one sitting. The retention rate for follow-up assessments was 80% (8/10). No significant changes in child participants’ diabetes self-management knowledge test scores, self-efficacy, parental communication, or HbA_1c_ levels were observed at 4- to 6-week follow-up (Table 4). Among parent participants, no significant changes were observed for the diabetes knowledge, attitudes, and behavioral measures. Although not statistically significant, we did find trends toward increasing knowledge and decreasing self-efficacy for diabetes management among both children and parents.

**Table 4:** Pre- and postchanges in knowledge, attitudes, and behaviors of 10 parent-child pairs participating in phase 2 of the Mobile Diabetes Educator pilot study (2018-2019).

Outcome		Pretest (N=10)	Posttest (N=8)	*P* value^a^
**Child**				
	Diabetes Knowledge Test score (percentage correct)^b^, mean (SD)	51.6 (21.8)	65.0 (9.5)	.08
	Child self-efficacy in diabetes self-management score^b^, mean (SD)	59.9 (11.6)	55.3 (17.6)	.51
	Children who report “always” talking with their parents when they have problems managing their diabetes^c^, n (%)	6 (60)	5 (63)	.35
	Children who report a score of 4 or a 5 for believing they can manage diabetes by themselves (high confidence)^c^, n (%)	2 (20)	3 (38)	.36
	Hemoglobin A_1c_ levels^b^, mean (SD)	9.6 (1.6)	9.9 (1.5)	.71
**Parent**				
	Diabetes Knowledge Test score (percentage correct)^b^, mean (SD)	60.0 (14.9)	70.8 (13.9)	.12
	Parental self-efficacy in helping their child manage diabetes score^b^, mean (SD)	78.5 (6.2)	70.5 (22.1)	.29
	Parents who report that their child “always” tells them when he or she is having problems managing diabetes^c^, n (%)	8 (80)	4 (50)	.50
	Parents who report a score of 4 or a 5 for believing that their child can manage diabetes on his or her own (high confidence)^c^, n (%)	4 (40)	3 (38)	.66
	Average number of times their child had a blood glucose checked in the last 24 hours^b,^ mean (SD)	4.4 (2.0)	3.6 (2.1)	.05

^a^*P* values are from paired *t* tests for continuous measures, and *P* values are from the Fisher exact test for categorical measures.

^b^Continuous measure.

^c^Categorical measure.

## Discussion

### Principal Findings

This pilot study demonstrated that the MDE was an acceptable and appropriate mobile health intervention for insulin-dependent children and their parents in a clinical setting. Participants were particularly satisfied with the overall layout of the tool (ie, mix of text, videos, and activities) and the information conveyed. The activities were often cited as the most engaging aspect of the tool and a positive way to reinforce the knowledge learned. Although the MDE was well received by participants, qualitative data indicated a need for improvements to the usability of the tool. Specifically, participants suggested adding directions to each slide and fixing malfunctioning activities. Participants also expressed a strong desire for a shorter duration.

Overall, no significant differences were observed between baseline and follow-up assessments among child and parent participants’ knowledge, attitudes, behaviors, and outcomes related to diabetes self-management. Given that this was a small feasibility study that was not powered to detect changes in outcomes, null findings were expected. With respect to our clinical outcome (child HbA_1c_ levels), null findings may also be because of the short follow-up period and the nature of the intervention. There is mixed evidence of educational and mobile-based interventions and their ability to affect HbA_1c_ in pediatric diabetes populations [8,10,28], especially during adolescence when glycemic control typically worsens [29]. Although not statistically significant, slight decreases in parental self-efficacy and the percentage of parents who perceived that their child could manage diabetes on their own were observed. One potential explanation for this finding is that the MDE highlighted gaps of knowledge and understanding of how to manage diabetes among parents, and the finding suggests that such tools may be used to help identify and target the lack of understanding or misperceptions of diabetes management among children and parents. We also observed a notable, although statistically insignificant, decrease in the percentage of parents who reported that their child *always* tells them when they are having problems managing diabetes. This trend could be the result of parent-child communication facilitated by the MDE about diabetes self-management that helped identify gaps in the child’s ability to manage diabetes.

### Lessons Learned

The findings from this mixed methods pilot study provide useful insight into the usability and acceptability of the MDE. Key recommendations regarding usability focused on directions and malfunctioning activities, whereas recommendations to improve acceptability focused on duration and animated characters. In regard to intervention delivery, we learned that youth with diabetes and their parents would also be interested in using this tool outside of the clinical setting (eg, at home via app). These lessons learned will be used to shape the next prototype of the MDE. A responsive website design, ideal for the burgeoning predominance of mobile Web browsing, could be included in the next iterative developmental stage along with translation to Spanish to further extend the target of this diabetes intervention tool to another high-risk underrepresented patient population.

### Comparison With Prior Work

Prior research demonstrates that mobile health interventions can be used to target knowledge, self-efficacy, and self-management behavior among patients with diabetes [30-32]. Characteristics of prior mobile health interventions for diabetes include *access to educational information* (eg, text pages, videos, and Web-based simulation), *health information storage* (eg, blood glucose readings and medication tracking), *social networking* (eg, storyboard, blog, and discussion board), and *communication* (eg, with health professionals, among parents and children) [12,15,30,31,33]. One key feature of our tool was the *activities* that engaged children in diabetes education (Figure 1).

Suggestions to improve mobile educational tools from our study are consistent with research on educational mobile tools for the self-management of other chronic diseases among youth. For example, adolescents with asthma suggested that *informative videos* covering asthma topics (eg, visual demonstration of inhaler technique) and *quizzes* be included in an asthma self-management app [34], two specific suggestions that were also voiced by participants in our user testing interviews. To date, mobile health intervention studies for patients with diabetes have largely focused on adolescents [10,14-17,31,32]. Our pilot study targeted a younger age range (5-14 years) as well as parents to explore the feasibility and acceptability of a mobile educational intervention among parent-child pairs. Additional content may need to be tailored for this younger population, such as school accommodations and communication, special events (eg, parties and camp), strategies for injection, and instructions about foods. Our sample also consisted of youth from low-income and racial minority populations, who experience disproportionately higher rates of T2D and diabetes-related adverse events [35,36] and are inadequately represented in diabetes intervention research. These sample characteristics are significant, given that the data gathered from this pilot study (eg, user testing feedback) will be used to further develop the MDE prototype, better ensuring that the needs and preferences of affected populations are reflected in the design of interventions.

### Strengths and Limitations

Strengths of this study include the in-depth qualitative methods used to examine intervention usability, acceptability, and feasibility and the recruitment of underserved study participants from an urban hospital setting to inform intervention refinement and study procedures for a larger trial. Limitations of this study include the small sample size, the absence of a control group for comparisons, a short follow-up period, and limited generalizability. Although it was a strength that our sample consisted of ethnically diverse, primarily low-income families, this limited our ability to control for race and ethnicity and income.

### Conclusions

Given the ubiquity of mobile devices, a child-centered mobile health intervention that engages children and parents has the potential to enhance pediatric diabetes management. The findings from this pilot study will be used to inform the next iteration of the MDE tool so that the user testing feedback can be incorporated and the intervention efficacy can be tested on a larger scale.

## Appendix

Multimedia Appendix 1 Semistructured interview guide.

## Abbreviations

BMC: Boston Medical Center
HbA_1c_: hemoglobin A_1c_
MDE: Mobile Diabetes Educator
T1D: type 1 diabetes
T2D: type 2 diabetes
