# Supplementary material for: Testing Usability and Feasibility of a Mobile Educator Tool for Pediatric Diabetes Self-Management: Mixed Methods Pilot Study
Source: JMIR Form Res. 2020 May 1;4(5):e16262. doi: 10.2196/16262 (PMC7229529; doi:10.2196/16262)
Supplement: Multimedia Appendix 1 [file formative_v4i5e16262_app1.docx]

**Multimedia Appendix 1.** Semi-structured interview guide

*Pre-amble:* Hi everyone! My name is _________. Thank you for participating in this interview. I’ll be asking you some questions about your experiences using the Mobile Diabetes Educator tool. Before we begin, I want to remind you what we say during this session will be confidential – your name will not be used, so please feel comfortable to share your thoughts. I will be taking a few notes and recording our conversation. This will help me remember what you say. You can ask me questions at any time. There are no right or wrong answers, and you do not have to answer questions if you do not wish to. Your answers are very important and can help researchers improve the tool. Do you have any questions before we get started?

**Usability (10 min.)**

This set of questions asks about how user-friendly the tool is. We will ask about the content later. For now, tell me about:

- How easy or difficult was it to use the tool?
- Did you have any problems figuring out how to use the tool? If so, tell me about them.
- What might make this tool more user-friendly?

**Acceptability (15 min.)**

Tell me about what you learned from the tool.

How easy or difficult was it to understand the material covered in each chapter?

- Which parts or chapters were the hardest to understand? Easiest?

What parts of the tool did you like the most? What made them fun or interesting?

- What did you think about the main character?
- What did you think about the settings?
- Which chapters or activities did you like the most? Why?

Which chapters or activities did you find less interesting? What made them less interesting?

What do you think the main purpose of the tool is?

**Overall Satisfaction (5 min.)**

How satisfied do you feel overall about the tool?

On a scale of 1-10, 10 being the best, how would you rate your satisfaction with the tool?

**Suggestions for Improvement (5 min.)**

What could make this tool better?

- What other information would you have wanted to learn more about?
- What other activities or chapters would you like the tool to include?

Any other thoughts about the tool?

Thank you so much for participating!
